# Defined Pig Microbiota Mixture as Promising Strategy against Salmonellosis in Gnotobiotic Piglets

**DOI:** 10.3390/ani14121779

**Published:** 2024-06-13

**Authors:** Nikol Modrackova, Kristyna Horvathova, Chahrazed Mekadim, Igor Splichal, Alla Splichalova, Ahmad Amin, Jakub Mrazek, Eva Vlkova, Vera Neuzil-Bunesova

**Affiliations:** 1 Department of Microbiology, Nutrition and Dietetics, Czech University of Life Sciences Prague, Kamycka 129, 165 00 Prague, Czech Republic; horvathovak@af.czu.cz (K.H.); ahmadamin@af.czu.cz (A.A.); vlkova@af.czu.cz (E.V.); bunesova@af.czu.cz (V.N.-B.); 2 Institute of Animal Physiology and Genetics of the Czech Academy of Sciences, Videnska 1083, 142 20 Prague, Czech Republic; mekadim@iapg.cas.cz (C.M.); kubino77@gmail.com (J.M.); 3 Laboratory of Gnotobiology, Institute of Microbiology, Czech Academy of Sciences, Doly 183, 549 22 Novy Hradek, Czech Republic; splichal@gnotobio.cz (I.S.); splichalova@gnotobio.cz (A.S.)

**Keywords:** bacterial consortium, bifidobacteria, lactobacilli, clostridia, bacilli, *Salmonella typhimurium*

## Abstract

**Simple Summary:**

*Salmonella typhimurium* is one of the most widespread enteric pathogens causing enterocolitis in warm-blooded animals worldwide. It is frequently spread within pig production and threatens not only primary hosts but consumers of pork meat as well. Overall, finding novel non-antibiotic agents is desirable in preventing salmonellosis outbreaks or reducing ongoing illness, where multi-strain probiotics are promising adepts. We tested a multi-strain bacterial mixture in the gnotobiotic piglet model to verify potential probiotic and anti-*Salmonella* properties in vivo. The bacterial consortium colonized the gut successfully and protected piglet hosts against *Salmonella* translocation to the blood system. Thus, the assembled bacterial mix has a promising potential for probiotic intervention in pig production management.

**Abstract:**

Probiotics are a potential strategy for salmonellosis control. A defined pig microbiota (DPM) mixture of nine bacterial strains previously exhibited probiotic and anti-*Salmonella* properties in vitro. Therefore, we evaluated its gut colonization ability and protection effect against *S. typhimurium* LT2-induced infection in the gnotobiotic piglet model. The DPM mixture successfully colonized the piglet gut and was stable and safe until the end of the experiment. The colon was inhabited by about 9 log CFU g^−1^ with a significant representation of bifidobacteria and lactobacilli compared to ileal levels around 7–8 log CFU g^−1^. Spore-forming clostridia and bacilli seemed to inhabit the environment only temporarily. The bacterial consortium contributed to the colonization of the gut at an entire length. The amplicon profile analysis supported the cultivation trend with a considerable representation of lactobacilli with bacilli in the ileum and bifidobacteria with clostridia in the colon. Although there was no significant *Salmonella*-positive elimination, it seems that the administered bacteria conferred the protection of infected piglets because of the slowed delayed infection manifestation without translocations of *Salmonella* cells to the blood circulation. Due to its colonization stability and potential protective anti-*Salmonella* traits, the DPM mixture has promising potential in pig production applications. However, advanced immunological tests are needed.

## 1. Introduction

The mammalian gut microbiome is considered a functional organ of the overall system of the host’s organism that is involved in substantial nutrient and drug metabolism, immunomodulation, stabilization of the gut mucosal barrier, and protection against pathogens [1,2,3]. Microbiome establishment with subsequent development is a critical phase of the young’s life [4,5] that is regulated by several factors, notably environmental [6]. If microbial balance is not preserved or is long-term disturbed by antibiotic effects, dysbiosis usually accompanied by a loss of microbial diversity can predispose to developing several unsolicited health problems, such as an onset of chronic inflammatory diseases [7]. Moreover, debilitated individuals may then be prone to outbreaks of fatal bacterial infections by pathogens to the detriment of beneficial microbes [8,9].

A dysbiotic gut could be a conducive environment for evolving *Enterobacteriaceae* members, which profit in these unfavorable conditions for other bacterial commensals [10,11,12]. For example, *Salmonella* spp. is a minacious pathogen entering the food chain, significantly threatening the overall health of the population, especially through the contamination of products of animal origin, including pork meat [13,14,15]. Specifically, non-typhoidal *Salmonella enterica* subsp. *enterica* serovar Typhimurium (*S. typhimurium*) is mostly dangerous for young or immunocompromised individuals, causing enterocolitis affecting the host’s distal ileum and colon [16,17]. *Salmonella* strains are commonly spread throughout the farm, and even suckling piglets are a risk source [18]. Stress and weaning, often accompanied by intestinal dysbiosis, are the ideal opportunities for the outbreak of salmonellosis in piglets [19,20].

There are several approaches to suppress salmonellosis infection in pigs. The probiotic intervention seems to be a promising supportive mitigation strategy [21,22], competing not only against pathogen attachment but also subsequent disease development [23]. Probiotics are life microorganisms whose administration in adequate amounts confers health benefits to the hosts [24]. In addition, thanks to all probiotic functional properties, they have the potential to replace some antibiotics [25,26], which are still widely administered, although there is a long-term approach to mitigate the spread of antibiotic resistance that has a far-reaching adverse effect on overall population health [27,28,29]. Furthermore, probiotics can exhibit antitoxin effects and contribute to an increase in growth performance [30]. Promisingly, the current trend of probiotics application in animal husbandry is still growing [31].

Recent studies pointed out several microbes with probiotic potential, promising for administration in swine production, such as lactobacilli [32], bifidobacteria [33], enterococci [34], *Bacillus* spp. [35], *Clostridium* spp. [36], and *Saccharomyces cerevisiae* [37]. Moreover, multi-strain probiotic mixtures could confer more complex effects with variable mechanisms, but it is crucial to focus on the evidence-based trials of probiotics efficacy [38]. Probiotic potential testing usually starts at the in vitro level with the subsequent implementation of in vivo assays to simulate the effect in complex host environments [39,40].

We previously developed a defined pig microbiota (DPM) mixture with in vitro-confirmed anti-*Salmonella* activity [41]. The aim of this work was to evaluate the colonization ability of this bacterial consortium and to verify its protective effect against salmonellosis induction, or the mitigation of induced infection, in vivo in gnotobiotic piglets by monitoring the clinical signs of infection and analyzing the ileal and colon contents by cultivation and microbiome analyses.

## 2. Materials and Methods

### 2.1. Ethical Approval

The work with animals was conducted according to the ethical standards defined by the EU legislation on the use of experimental animals (2010/63/EU) and approved by the Animal Care and Use Committee of the Czech Academy of Sciences (protocol 57/2021; 18 August 2021).

### 2.2. Design of In Vivo Experiment

**Figure 1:**
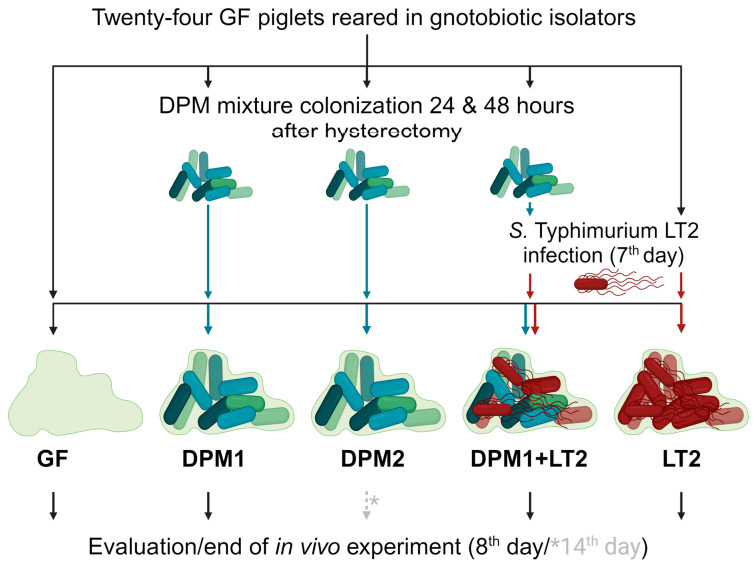
Schema of in vivo experiment. Gnotobiotic piglets (*n* = 24) were divided into five experimental groups and were kept for two experimental time periods (8 days/14 days). After 24 and 48 h after hysterectomy, three groups (DPM1, DPM2, and DPM1 + LT2) were orally colonized by the defined pig microbiota mixture. On the seventh day of the piglets’ life, two groups (DPM1 + LT2 and LT2) were infected with *S. typhimurium* LT2. The eighth day was the end of the in vivo experiment for all groups with the exception of the DPM2 group, which ended on the fourteenth day. GF—germ-free; DPM—defined pig microbiota; DPM1—piglets associated with DPM mixture for 8 days; DPM2—piglets associated with DPM mixture for 14 days; DPM1 + LT2—piglets associated with DPM mixture and infected with *S. typhimurium* LT2; LT2—piglets infected with *S. typhimurium* LT2.

Germ-free (GF) piglets were obtained by the hysterectomy of pregnant miniature sows (Animal Research Institute, Kostelec nad Orlici, Czech Republic) under isoflurane anesthesia, and they were reared in gnotobiotic isolators and fed an autoclave-sterilized cow milk-based diet (CMD) with mineral and vitamin supplements [42]. Twenty-four gnotobiotic piglets were divided into five experimental groups: GF (GF piglets; negative control), LT2 (piglets infected with *S. typhimurium* LT2; positive control), DPM1 (piglets colonized by DPM mixture for 8 days), DPM2 (piglets colonized by DPM mixture for 14 days), and DPM1 + LT2 (piglets colonized by DPM mixture and then infected with *S. typhimurium* LT2) (Figure 1).

In general, GF piglets were repeatedly orally colonized with a single dose of DPM mixture (previously stored at −80 °C) in a CMD containing 6 × 10^8^ CFU, 24 h and 48 h after hysterectomy. Then, seven-day-old piglets were orally infected by 1 × 10^6^ CFU mL^−1^ of *S. typhimurium* LT2 in the CMD, as well [43]. Twenty-four hours after the *Salmonella* infection, the experiment was terminated for all groups except the DPM2 group, whose time was prolonged by the next six days (termination on their fourteenth day) for monitoring the DPM mixture’s safety over time and colonization stability. All animals were examined for clinical signs of enterocolitis (fever, anorexia, somnolence, and diarrhea) during the whole experiment. Finally, all subjects were exsanguinated via cardiac puncture under isoflurane anesthesia and samples, such as ileum and colon contents, mesenteric lymph nodes, and blood, were taken for cultivation and microbiome analysis at the end.

### 2.3. Bacteria Propagated for Association/Infection of Gnotobiotic Piglets

A DPM mixture consisting of nine bacterial strains, namely *Bacillus* sp. PG1, *Bifidobacterium animalis* subsp. *lactis* PG2, *B. porcinum* PG3, *Clostridium sporogenes* PG4, *Lactobacillus amylovorus* PG6, *L. paracasei* subsp. *tolerans* PG5, and three *Limosilactobacillus reuteri* strains (PG7, PG8, PG9), was prepared as described by Horvathova et al. [41]. These strains have previously exhibited anti-*Salmonella* activity, the ability to aggregate, adherence to epithelial cells, and bile and acid tolerance, and they have been without mutual inhibition and classified as safe without a pathogenic phenotype and resistance to antibiotics in vitro.

The *S. typhimurium* LT2 inoculum [44] was prepared and 1 × 10^6^ CFU mL^−1^ per piglet was orally applied in 1 mL of CMD as described previously Splichal et al. [45]. The used dose was verified by cultivation overnight at 37 °C on Brilliant Green agar (Oxoid, Basingstoke, UK).

### 2.4. Cultivation Analysis

Ileum and colon content, mesenteric lymph nodes, and blood samples were analyzed for the presence of administered pig microbiota and *S. typhimurium* LT2 according to Modrackova et al. [46]. All samples were collected in tubes containing a dilution buffer consisting of 5 g L^−1^ of tryptone, 5 g L^−1^ of nutrient broth No. 2, 2.5 g L^−1^ of yeast extract (all Oxoid), 0.5 g L^−1^ of L-cysteine, 1 mL L^−1^ of Tween 80 (both Sigma-Aldrich, St. Louis, MO, USA), 30% glycerol (VWR, Radnor, PA, USA), and glass pearls for homogenization. All media were prepared in an oxygen-free carbon dioxide environment [47] and then sterilized. Immediately after dissection and sampling, all samples were frozen at −20 °C and transported to the analysis site within the same week. Then, decimal serial dilutions were spread on the following media. Wilkins-Chalgren Anaerobe Agar supplemented with 5 g L^−1^ of GMO-Free Soya Peptone (both Oxoid), 0.5 g L^−1^ of L-cysteine, and 1 mL L^−1^ of Tween 80 was used to determine the total counts of anaerobic bacteria (WSP). The same WSP medium was used for heat-resistant spore-forming bacteria (clostridia/bacilli group) as well; however, the inocula were previously treated with 15 min of pasteurization at 80 °C before plating. Then, the following two WSP medium variants were used for bifidobacteria: WSP supplemented with 100 mg L^−1^ of mupirocin (Oxoid) and 1 mL L^−1^ of acetic acid (Sigma-Aldrich) [48] (WSP-MUP), and WSP supplemented with 100 mg L^−1^ of mupirocin, 100 mg L^−1^ of norfloxacin (Oxoid), and 1 mL L^−1^ of acetic acid [49] (WSP-NORF). All the plates were incubated anaerobically using GENbag anaer (bioMérieux, Craponne, France) at 37 °C for 2 days. Then, Rogosa Agar (Oxoid) with 1.32 mL L^−1^ of acetic acid was used for the quantification of lactobacilli with subsequent cultivation under microaerophilic conditions at 37 °C for 3 days, and SS agar (Oxoid) for *S. typhimurium* LT2 under aerobic conditions at 37 °C for 24 h. An identity of determined bacteria was always verified with MALDI-TOF mass spectrometry (MALDI-TOF MS) using an extended direct transfer procedure with an HCCA matrix solution (α-cyano-4-hydroxycinnamic acid) according to the manufacturer’s instructions (Bruker Daltonik GmbH, Bremen, Germany) using Biotyper software (server distribution version 4.1.100 (PYTH), build 174; server module version 4.3.18, build 330).

#### Statistical Analyses of Cultivation Data

The bacterial mixture stability in vivo in time and its potential protective effect against *S. typhimurium* LT2 infection in gnotobiotic piglets (cultivation counts in log CFU g^−1^) were evaluated using StatSoft, Inc. (2013) (STATISTICA—data analysis software system, version 12, www.statsoft.com (accessed on 18 January 2024); Tulsa, OK, USA) and MS Excel 2013 (Redmond, WA, USA). The normality of the data was assessed by the Shapiro–Wilk W test (α = 0.05). Differences between the levels of lactobacilli, bifidobacteria, and clostridia/bacilli within the ileum and colon and two colonization time durations (DPM1, DPM2 animal groups) were evaluated using a *t*-test (α = 0.05). Then, differences in bacterial counts between DPM1 and DPM1 + LT2 groups, and similarly DPM1 + LT2 and LT2 groups, within the ileum and colon separately were assessed using a *t*-test (α = 0.05) and a Mann–Whitney U Test (α = 0.05).

### 2.5. 16S rRNA Gene Amplicon Sequencing

Total genomic DNA was extracted from 250 mg of ileum and colon fecal content using the QIAamp^®^ PowerFecal^®^ Pro DNA Kit (Qiagen, Hilden, Germany) according to the manufacturer’s instructions. The extracted DNA was then used as a template for the preparation of amplicons from the V4 region of the 16S rRNA gene [50]. Purified amplicons were used to prepare libraries using the NEBNext Fast DNA Library Prep Set kit (New England Biolabs, Ipswich, MA, USA) according to Milani et al. [51]. The sequencing was then performed on the PGM sequencer of the Ion Torrent platform (Thermo Fisher Scientific, Waltham, MA, USA) as it was described previously by Mekadim et al. [52].

### 2.6. Microbiome Analyses

Analysis of the obtained sequences was carried out using the QIIME 2 version 2022.2 pipeline [53]. Raw sequences were filtered and trimmed using DADA2 [54]. Then, the obtained amplicon sequence variants (ASVs) were taxonomically classified using VSEARCH based on the SILVA database (release 138) with a 99% threshold [55]. The rarefaction was performed based on the sequence depth to normalize data. The alpha diversity of different animal groups was determined using Chao1, Shannon, and Simpson diversity indexes based on the Kruskal–Wallis test. Principal Coordinate Analysis (PCoA) was evaluated based on the weighted and unweighted unifrac distance (beta diversity). The boxplots for alpha diversity and the two-dimensional PCoA plots were generated in R-Studio (http://www.rstudio.com/, accessed on 16 October 2023) using qiime2R (https://github.com/jbisanz/qiime2R, accessed on 16 October 2023) and ggplot2 (https://ggplot2.tidyverse.org, accessed on 16 October 2023) packages. Permutational multivariate analysis of variance (PERMANOVA/Adonis) based on distance matrices was used to evaluate the dissimilarity among animal groups with the permutation set at 999. Linear discriminant analysis with the effect size (LEfSe) algorithm [56] was performed in the Galaxy module (http://huttenhower.sph.harvard.edu/galaxy (accessed on 18 October 2023) to identify bacterial genera with significant differential relative abundances between animal groups based on the Kruskal–Wallis test and the pairwise Wilcoxon test with an α value of 0.05 and threshold value at 2.0.

### 2.7. Data Accessibility

Sequences of raw data files were deposited in the NCBI database under Sequence Read Archive (SRA) accession numbers SUB13949577 and BioProject ID PRJNA1035042.

## 3. Results

To evaluate the ability of the DPM mixture to colonize the gut of piglets, namely the ileum and colon, and its possible protective effect against the infection caused by *S. typhimurium* LT2, the cultivation analysis and amplicon sequencing approaches were combined. In general, gnotobiotic piglets were divided into five monitored groups with different schemes of bacterial associations for the assessment of various DPM mixture’s abilities. The GF control animal group receiving no bacteria and only sterile CMD throughout the experiment confirmed the gnotobiotic model assay with both approaches.

### 3.1. Colonization Ability of DPM Mixture in Gnotobiotic Piglets

The colonization ability of the DPM mixture and its potential competitive capability against *S. typhimurium* LT2 were assessed in DPM1 + LT2, DPM1, and LT2 animal groups within two parts of the piglet gut, the ileum and colon. The detected high quantity of viable bacteria present in both parts of the gut confirmed the success of colonization of all bacteria from the administered bacterial consortium (Supplementary Material—Table S1). We detected no statistically significant differences in bacterial representation (lactobacilli, clostridia/bacilli, and *S. typhimurium* LT2) between the monitored experimental groups, except for bifidobacteria. Specifically, bifidobacterial counts on WSP-NORF were significantly higher in DPM1 (7.56 ± 0.14 log CFU g^−1^) compared to the DPM1 + LT2 (6.90 ± 0.63 log CFU g^−1^) group in the ileum, and on WSP-MUP in DPM1 (9.38 ± 0.23 log CFU g^−1^) compared to DPM1 + LT2 (8.43 ± 0.77 log CFU g^−1^) in the colon. However, based on the cultivation results, the infection caused by *S.* Typhimurium LT2 did not significantly influence other prior administered bacteria and vice versa.

Comparing the bacterial consortium colonization ability of two different parts of the piglets’ gut, our results showed that the DPM mixture successfully colonized both (Supplementary Material—Table S2). In general, the colon was considerably more colonized by administered bacteria in density more than 10^9^ CFU g^−1^ of total counts of bacteria in all monitored experimental groups, while the bacteria present in the ileum reached lower concentrations of 10^7^–10^8^ CFU g^−1^. Specifically, the lactobacilli (9.13 ± 0.50 log CFU g^−1^) and bifidobacterial (9.38 ± 0.23 log CFU g^−1^ on WSP-MUP; 8.94 ± 0.13 log CFU g^−1^ on WSP-NORF) levels were significantly higher in the colon compared to a representation about an order of magnitude lower in the ileum within the DPM1 group. Similarly, the same significant trend was found for *S. typhimurium* LT2 within the DPM1 + LT2 (9.08 ± 0.48 compared to 8.27 ± 0.55 log CFU g^−1^) and LT2 (8.61 ± 0.05 compared to 7.73 ± 0.37 log CFU g^−1^) groups. Although not statistically significant, this difference was evident also for other bacterial groups.

### 3.2. Alpha and Beta Diversity of the Piglet’s Gut Microbiome

**Figure 2:**
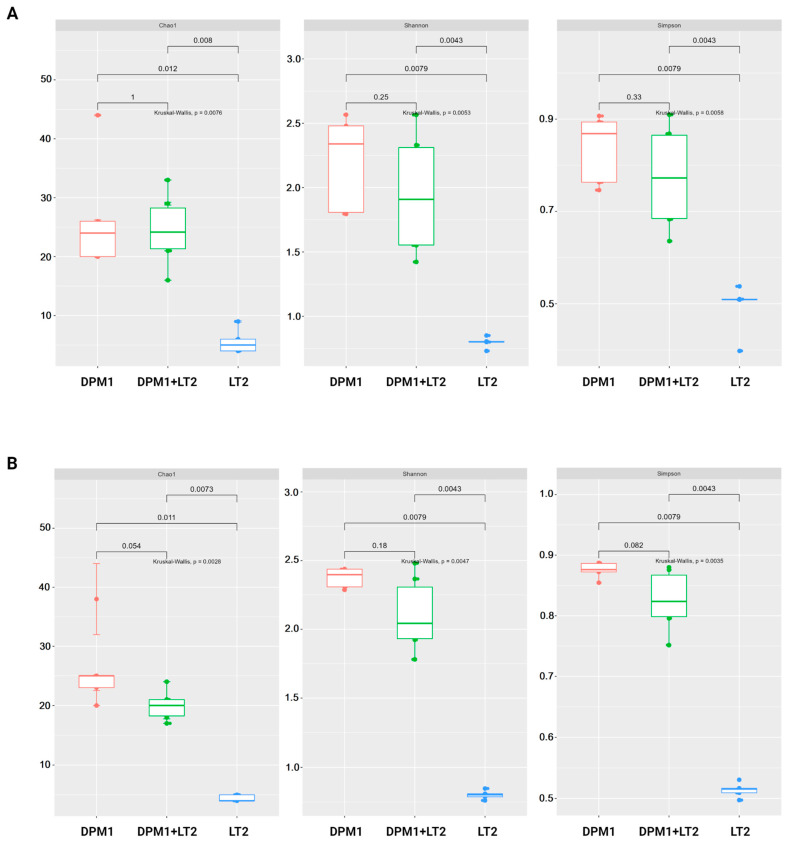
Alpha diversity of the piglets’ gut microbiome. Boxplots illustrating alpha diversity using Chao1, Shannon, and Simpson diversity indices in the (**A**) ileum and (**B**) colon of DPM1, DPM1 + LT2, and LT2 animal groups. *p* ≤ 0.05 was considered statistically significant based on the Kruskal–Wallis test. DPM1—piglets associated with DPM mixture for 8 days; DPM1 + LT2—piglets associated with DPM mixture and infected with *S. typhimurium* LT2; LT2—piglets infected with *S. typhimurium* LT2.

The results of alpha diversity using Chao1, Shannon, and Simpson diversity indexes are represented in the boxplot graph (Figure 2). In both the colon and ileum, bacterial diversity and richness were significantly higher in DPM1 and DPM1 + LT2 animal groups in comparison to the LT2 group (*p* ≤ 0.05). No significant difference was observed between DPM1 and DPM1 + LT2 groups in both intestinal parts (ileum and colon).

**Figure 3:**
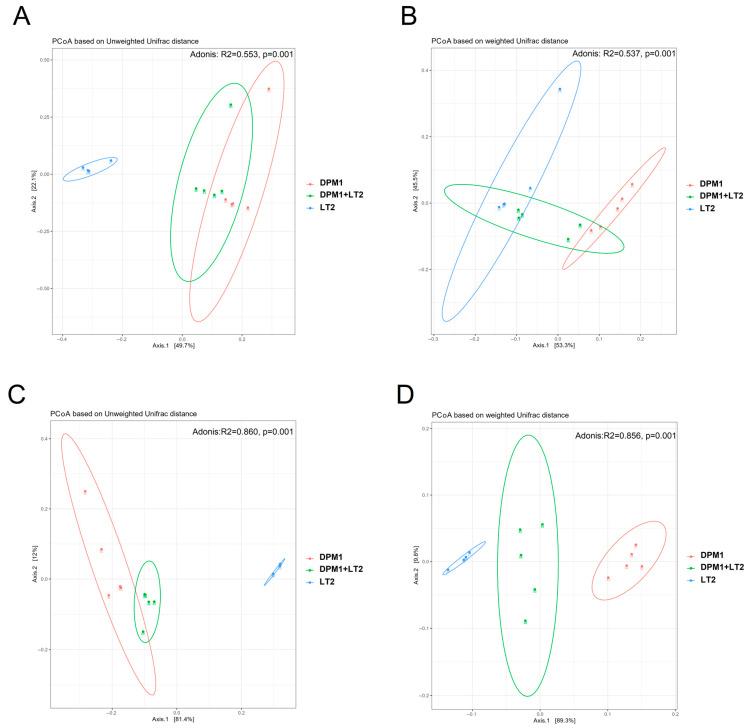
Comparison of piglets’ microbiome beta bacterial diversity. The beta diversity using Principal Coordinate Analysis (PCoA) plots based on the unweighted (**A**,**C**) and weighted (**B**,**D**) unifrac distance showed distinct clusters of bacterial populations of the (**A**,**B**) ileum and (**C**,**D**) colon from DPM1, DPM1 + LT2, and LT2. *p* ≤ 0.05 was considered statistically significant. DPM1—piglets associated with DPM mixture for 8 days; DPM1 + LT2—piglets associated with DPM mixture and infected with *S. typhimurium* LT2; LT2—piglets infected with *S. typhimurium* LT2.

Principal Coordinate Analysis (PCoA) based on weighted and unweighted unifrac distances was performed to compare the bacterial diversity in the ileum and colon of DPM1, DPM1 + LT2, and LT2 animal groups (Figure 3). Both the colon and ileum presented distinguished and separated clusters showing a profound difference in microbiome diversity (*p* = 0.001) between different animal groups. Using the unweighted unifrac distance, the cluster of samples from LT2 animals was regrouped together and separated from the other clusters of DPM1 and DPM1 + LT2 groups that were clustered close.

**Figure 4:**
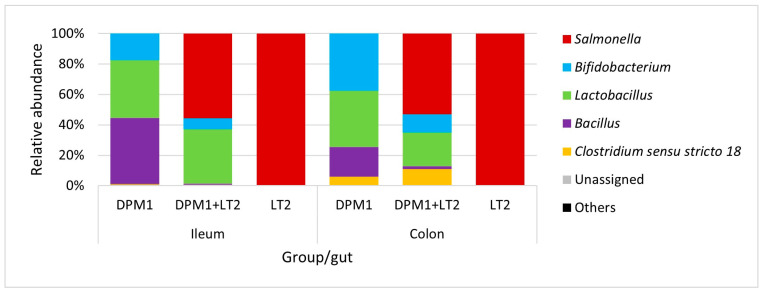
Relative abundance of present bacteria in ileum and colon at the genus level. DPM1—piglets associated with DPM mixture for 8 days; DPM1 + LT2—piglets associated with DPM mixture and infected with *S. typhimurium* LT2; LT2—piglets infected with *S. typhimurium* LT2.

*Bacillus*, *Lactobacillus*, and *Bifidobacterium* were the most abundant genera in the ileum of DPM1 animals (Figure 4). We observed an increase in the abundance of *Bifidobacterium* spp. and *Clostridium* sensu stricto 18 and a decrease in the abundance of *Bacillus* spp. in the colon microbiota of the DPM1 animals. *Salmonella* was the dominant genus in the ileum microbiota of DPM1 + LT2. *Lactobacillus* spp. and *Bifidobacterium* spp. were also present in the ileum microbiota of the DPM1 + LT2 group. The abundance of *Bifidobacterium* spp. and *Clostridium* sensu stricto 18 was increased and the abundance of *Lactobacillus* spp. was decreased in the colon microbiota of the DPM1 + LT2 group with a dominance of *Salmonella* spp. *Salmonella* was the only bacterial genus present in both ileum and colon microbiota in the LT2 animal group.

**Figure 5:**
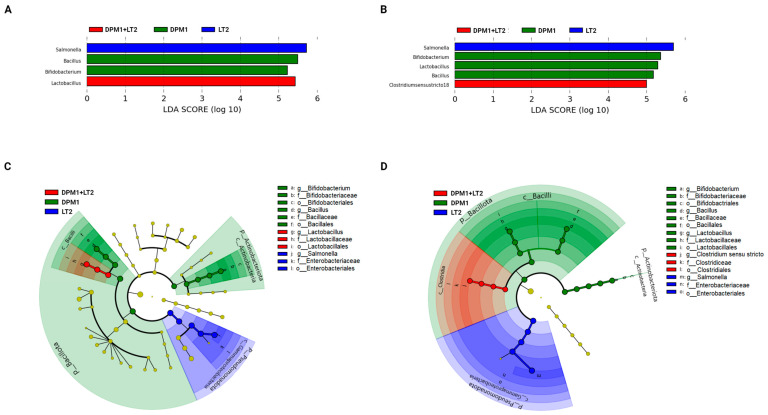
Detected bacterial genera as biomarkers. Linear discriminant analysis effect size (LEfSe) of taxa at the genus level in the bacterial community of the (**A**) ileum and (**B**) colon of DPM1, DPM1 + LT2, and LT2 animal groups with alpha values of 0.05 and a threshold value of 2.0. [(**C**,**D**) are cladograms presenting a phylogenetic plot of LEfSe at different taxonomical levels of bacterial community in the (**C**) ileum and (**D**) colon of DPM1, DPM1 + LT2, and LT2 animal groups]. DPM1—piglets associated with DPM mixture for 8 days; DPM1 + LT2—piglets associated with DPM mixture and infected with *S. typhimurium* LT2; LT2—piglets infected with *S. typhimurium* LT2.

Linear discriminant analysis with effect size (LefSe) was used to detect bacterial genera (biomarkers) with significant differential relative abundances in ileum and colon microbiomes of animal groups (Figure 5). In the ileum, *Bacillus* and *Bifidobacterium* were the bacterial genera markers of the DPM1 animal groups. *Lactobacillus* was the only biomarker for DPM1 + LT2 pigs, while, in the colon, *Bifidobacterium*, *Lactobacillus*, and *Bacillus* were bacterial markers of the DPM1 animal groups. *Clostridium* sensu stricto 18 was the only bacterial genera marker of DPM1 + LT2 pigs. *Salmonella* was the unique biomarker of the infected LT2 animals in both ileum and colon microbiomes.

### 3.3. Assessment of a Possible Protective Effect of the Bacterial Mixture In Vivo

To assess the possible protection ability of the administered DPM mixture, all piglet groups were monitored for the clinical signs of infection. Piglet groups DPM1, DPM2, GF, and DPM1 + LT2 thrived for the whole experiment. Although DPM1 + LT2 individuals visually prospered, as well, there were detected typical clinical signs of the enteric infection such as fever, anorexia, sleepiness, and diarrhea; however, in decreased manifestations compared to the control LT2 group, in which the clinical signs initially appeared 6 h after infection. Moreover, cultivation analysis of blood samples showed the absence of bacteria within the DPM1, DPM2, and DPM1 + LT2 groups, while the presence of bacteria, specifically *S*. *typhimurium* LT2, in the blood was positive within the LT2 group (4.68 ± 0.68 CFU mL^−1^). Moreover, the bacterial presence in the mesenteric lymph nodes was confirmed, namely 4.86 ± 0.27 CFU g^−1^ for DPM1 + LT2 and 5.49 ± 0.43 CFU g^−1^ for LT2 groups.

### 3.4. Evaluation of Bacterial Consortium Stability and Safety in Time

**Figure 6:**
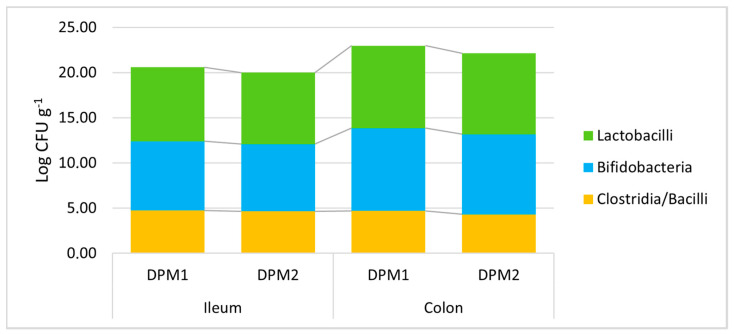
Stability of DPM mixture in the piglets’ gut in time. DPM1—piglets associated with DPM mixture for 8 days; DPM2—piglets associated with DPM mixture for 14 days.

Additionally, the stability of the DPM mixture and the possible risk of pathobiont outbreak after a longer colonization time in vivo were assessed. Piglets in the DPM2 group were monitored under the same in vivo model conditions for 14 days and were compared to the DPM1 group. All determined viable bacterial levels did not significantly change after a prolongated time of the in vivo experiment in both parts of the gut (Figure 6, Supplementary Material—Table S3). In comparison with the concentration of approximately 5.50 × 10^7^ CFU mL^−1^ of each bacterial strain in the administered mixture, we detected slightly higher numbers of lactobacilli and bifidobacteria compared to numbers of heat-resistant spore-forming bacteria about three orders of magnitude lower (clostridia and bacilli).

Furthermore, a slight non-significant trend of decreasing bacterial levels was found for longer periods of colonization. Then, there was a similar confirmation of the significant trend of higher bacterial levels in the colon environment compared to the ileum for lactobacilli and bifidobacteria (Supplementary Material—Table S4). Moreover, no pathobiont outbreak was detected; thus, the safety of the mixture described in vitro was also confirmed in vivo. Furthermore, a possible long-term colonization could be expected for the analyzed DPM mixture.

### 3.5. Piglets’ Gut Microbiome Alpha and Beta Diversity in Time

**Figure 7:**
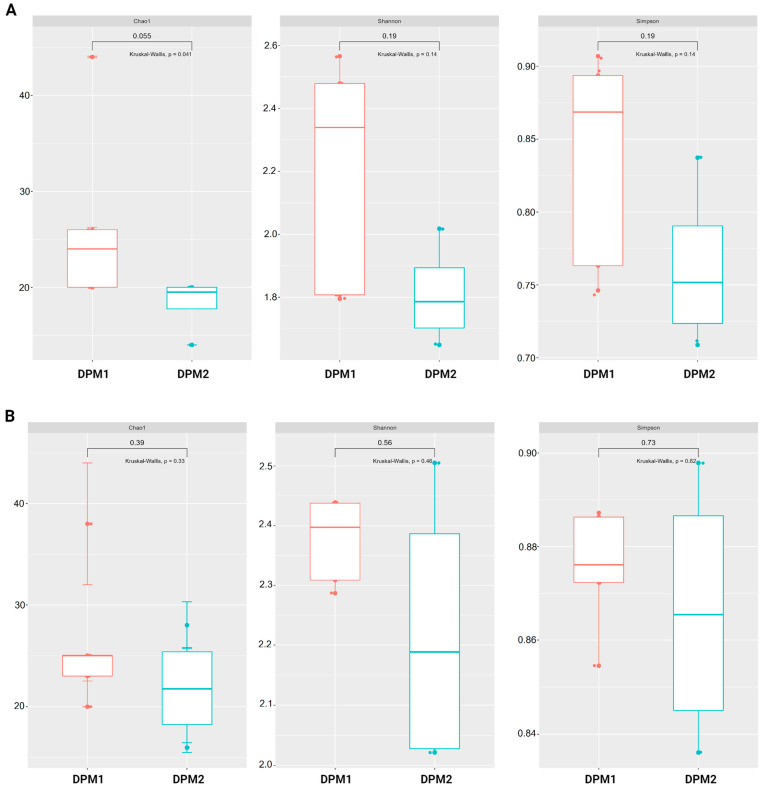
Effect of the in vivo experimental time on alpha diversity of the gut microbiome. Boxplots illustrating alpha diversity using Chao1, Shannon, and Simpson diversity indices in the (**A**) ileum and (**B**) colon of DPM1 and DPM2 animal groups. *p* ≤ 0.05 was considered statistically significant based on the Kruskal–Wallis test. DPM1—piglets associated with DPM mixture for 8 days; DPM2—piglets associated with DPM mixture for 14 days.

**Figure 8:**
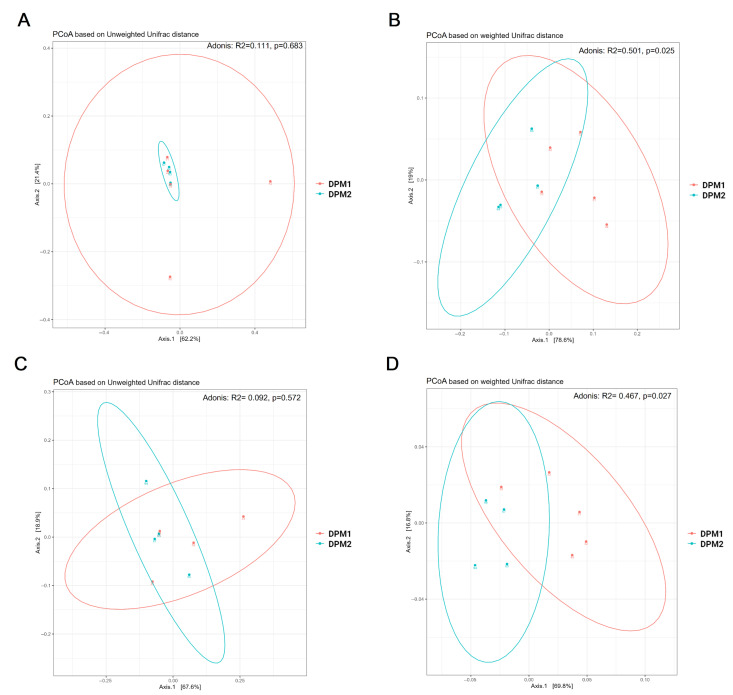
Bacterial beta diversity of gut microbiome in time. Beta diversity using Principal Coordinate Analysis (PCoA) plots based on the unweighted (**A**,**C**) and weighted (**B**,**D**) unifrac distance in the (**A**,**B**) ileum and (**C**,**D**) colon from DPM1 and DPM2 animal groups. *p* ≤ 0.05 was considered statistically significant. DPM1—piglets associated with DPM mixture for 8 days; DPM2—piglets associated with DPM mixture for 14 days.

Using Chao1, Shannon, and Simpson diversity indexes, no significant difference in alpha diversity was observed between DPM1 and DPM2 animal groups in both the ileum and colon microbiomes (*p* > 0.05) (Figure 7). The beta diversity of both colon and ileum microbiomes showed no significant difference between DPM1 and DPM2 animal groups using unweighted unifrac distances (*p* > 0.05) (Figure 8). However, a significant difference was observed in both colon and ileum microbiomes using weighted unifrac distances (*p* ≤ 0.05). That means that the difference between DPM1 and DPM2 was in the abundance and not in the composition.

**Figure 9:**
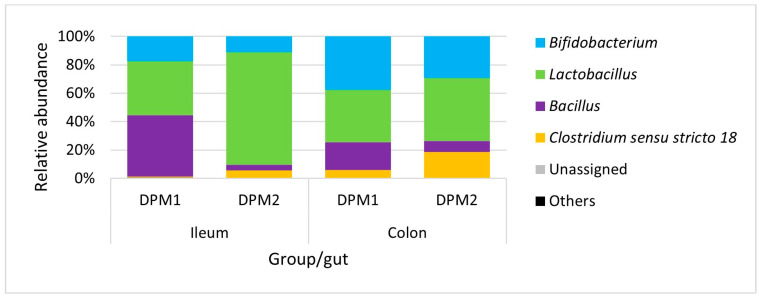
Relative abundance of present bacteria in time at the genus level. DPM1—piglets associated with DPM mixture for 8 days; DPM2—piglets associated with DPM mixture for 14 days.

Analyzing microbiome composition (Figure 9), *Lactobacillus* was the dominant genus in the ileum of DPM2 animals. The proportion of *Lactobacillus* was higher in the ileum microbiota of DPM2 in comparison to the ileum microbiome of DPM1 animals. *Bifidobacterium* and *Bacillus* were higher in the ileum microbiome of DPM1 animals. *Bifidobacterium*, *Bacillus*, and *Clostridium* sensu stricto 18 were also the identified abundant genera in the ileum microbiota of DPM2 animals. The abundance of *Bifidobacterium* and *Clostridium* sensu stricto 18 was increased in the colon microbiota of the DPM1 and DPM2 animals. The abundance of *Lactobacillus* was decreased only in the colon microbiota of the DPM2 animals.

Using LefSe analysis, no significant difference in relative abundance was observed between DPM1 and DPM2 in both ileum and colon microbiomes.

## 4. Discussion

Microbes inhabiting the pigs’ gut form a complex ecosystem conferring pivotal nutritional, physiological, and immunological functions for the host [57], such as the utilization of undigested substrates with various metabolic regulations [58], bioactive compound production [59], protection against pathogens [60], and immunostimulation [61]. Its diversity increases from birth to weaning and is significantly influenced by the environment and contact with sows [62]. Unfortunately, the pig farm environment is closely related to bacterial pathogens as well, specifically *S. typhimurium* which is the most frequent causative agent of infection development [63].

Salmonellosis outbreak significantly disrupts the autochthonous microbiota and affects the immune response [64]. Moreover, it threatens not only the life of the host but there is a significant risk of developing the disease in pork consumers, notably menacing the health of the global population [65] as a significant food safety hazard. Thus, it is desirable to increase the detection of *S. typhimurium* originating from farms in pork meat and ensure biosecurity precautions [66]. Positively, some countries have already developed several confident control measures for the management of the reduction in or even elimination of *Salmonella* exposition in the pig farms and pork production chain, e.g., the monitoring of animal movement and possible contacts; the treatment of feed and water; regular sampling at the animal, feed, and environment level; and thorough cleaning and disinfection [14,67,68], to avoid the recycling of this zoonotic hazard at the farm level.

In the era of antibiotic resistance spread in animal husbandry, there is a need to search for novel non-antibiotic alternatives to keep individuals in good health shape, where one of the promising strategies for pigs is a probiotic intervention [69,70,71]. Probiotic supplements in animal feed have already been used not only to improve the health parameters and welfare of the hosts, but also to reduce the action of alimentary pathogens as well [21]. Popularly, probiotics consisting of more bacterial strains could synergistically enhance the targeted effect [72], but it is important to perform strain selection based on the function and efficacy [38]. Moreover, there used to be a recommendation to use microbial strains of the same origin as the targeted host [73]. Thus, the pig origin of our administered multi-strain bacterial consortium, consisting of *B. animalis* subsp. *lactis* PG2, *B. porcinum* PG3, *L. amylovorus* PG6, *L. paracasei* subsp. *tolerans* PG5, *L. reuteri* PG7, PG8, PG9, *C. sporogenes* PG4, and *Bacillus* sp. PG1, probably contributed to the detected colonization success in the gnotobiotic piglets. Moreover, this combination of various bacterial species in one administered consortium conferred integral colonization of the gut, showing amplicon profiling results—specifically, a more considerable representation of lactobacilli and bacilli levels in the ileum and bifidobacterial with clostridial concentrations in the colon. In addition, the DPM mixture, assessed as safe, seems to have long-term colonization capacity.

In general, the pigs’ gut microbiota is subjected to dynamic shifts and is being changed during the life of the pig hosts, but *Bacteroides* spp., *Prevotella* spp., and *Lactobacillus* could be identified as their core bacterial genera [74]. Furthermore, bifidobacteria are considered highly abundant commensals in the gut of conventional pigs, and wild boars as well [75,76]. Similarly, clostridia [77,78] and bacilli [79] considerably occur in this complex environment. When focusing on the mentioned bacterial groups in the pig fecal microbiota, we previously confirmed their common occurrence [41]. Thanks to the fact that these commensals could be easily cultivated under the laboratory conditions and acquired selected isolates exhibited anti-*Salmonella* activity and probiotic potential in vitro, we combined the cultivation and amplicon sequencing approaches for in vivo assessment of the DPM mixture colonization ability in the gnotobiotic piglets’ model. Our results showed that the DPM mixture commendably colonized the gut of piglets with a significantly more inhabited colon compared to the ileum. It is generally recognized that colon microbiota is a mostly populated ecosystem within mammalian individuals with a detrimental impact on the host’s health and confers significant functions [80]. This bilateral, dynamic, and symbiotic relationship is enabled by the gut providing a favorable environment full of energy supplies and thriving conditions for microorganisms, securing eubiosis and creating the host–gut microbiota axis [81].

As follows, to verify the DPM mixture’s protective effect against *S. typhimurium* LT2-induced infection, we used the gnotobiotic piglet model [42]. Although the *Salmonella* load was not significantly decreased by supplementation with a consortium of nine bacterial strains in the gut of DPM1 + LT2, milder and slowed infection manifestations were detected in comparison with the LT2 control group. Moreover, administered commensals seem to interact with *S.* Typhimurium LT2, causing a protective barrier function against its entrance into the blood circulation, because no bacteria were detected in blood samples of the DPM1 + LT2 group compared to their presence in the LT2 group, where the pathogen translocation into the blood circulation was detected. Although there was a bacterial absence in the blood of the DPM1 + LT2 group, microbes were detected in their mesenteric lymph nodes and further interactions with *S. typhimurium* LT2 could be expected. These findings suggest the DPM mixture’s possible protective character that could probably confer to piglets’ immune stimulation. Interestingly, the in vivo amelioration of non-typhoidal *Salmonella* infection by supplementation with single probiotic strain *L. rhamnosus* GG has already been described before [43], as well as by *L. reuteri* KUB-AC5 [82], *B. animalis* subsp. *lactis* NFBAL23 [83], *B. thermophilum* RBL67 [84], and *E. coli* Nissle 1917 [45] or the in vitro anti-*Salmonella* activity of *L. amylovorus* SLZX20-1 [85] and *L. paracasei* DUP-13076 [86]. However, no anti-inflammatory reaction was revealed for single commensal lactobacilli strains, namely *L. amylovorus* P1 and *L. mucosae* P5. Interestingly, the authors highlighted a better protective potential when using multiple strains in a mixture [45]. Myhill et al. [87] support this suggestion showing two probiotic mixtures as well, namely *Bac. amyloliquefaciens* 516, *Bac. subtilis* 541 with *Enterococcus faecium* 669, and *B. animalis* subsp. *lactis* BB-12 with *L. rhamnosus* LGG, affecting the host’s immune homeostasis desirably.

Although bifidobacteria and lactobacilli are considered safe and are widely used as probiotics in variable food- and feedstuffs [88], there is a growing trend for looking for other applicable bacterial species for probiotic interventions in the form of vegetative cells or spores. In general, some species of spore-forming bacteria, such as clostridia and bacilli, are significant parts of the commensal gut microbiota and could confer several probiotic and technological benefits, e.g., anti-inflammation effects, sporulation behavior with thermostability, and the production of desired metabolites [89,90,91,92]. However, some of them remain controversial [93] and further research is necessary. Deng et al. [94] emphasize the necessity of thorough testing, as they detected hemolytic activity, carried enterotoxin and antibiotic resistance genes, and cytotoxicity in several analyzed probiotic supplements containing bacterial spores. However, based on our in vitro testing [41], we found clostridial and *Bacilli* spore-forming strains lacking these traits and showing promising probiotic character. Although their presence in the gut content from both parts was the lowest (10^3^–10^5^ CFU g^−1^) compared to other administered bacterial groups at the end of the in vivo experiment, it seems that they are part of the transient gut microbiota as previously described by Zhang et al. [95], but they probably contributed to the hosts’ well-being as well. The probiotic effect of several *Clostridium* and *Bacillus* strains has already been described before, e.g., belonging to species of *C. butyricum* [96], *Bac. subtilis* [97], *Bac. licheniformis* and *Bac. pumilus* [98], and *Bac. coagulans* [99].

The DPM mixture including viable bifidobacteria and lactobacilli and the heat-resistant *C. sporogenes* PG4 and *Bacillus* sp. PG1 have a promising probiotic potential for administration to pigs verified at the gnotobiotic piglet model in vivo thanks to its colonization stability, safety, and possible protective traits such as alleviating infection manifestation and preventing the translocation of *Salmonella* cells to the blood circulation. Nevertheless, further advanced tests are needed for the verification of its suitability for application in pig production.

## 5. Conclusions

Our results confirmed the colonization ability and potential protective effect of the administered multi-strain DPM mixture against *S. typhimurium* LT2. However, there is a need to carry out further tests, especially immunological ones. If the potential protective effect is confirmed, it is necessary to continue with in vivo testing with conventional pig hosts for observing the DPM mixture supplementation effects in natural husbandry conditions.

## Data Availability

The original contributions presented in the study are included in the article/Supplementary Material, and further inquiries can be directed to the corresponding author.

## Supplementary Materials

The following supporting information can be downloaded at https://www.mdpi.com/article/10.3390/ani14121779/s1. Table S1: Comparison of determined cultivation counts (log CFU g^−1^) of administered bacteria in the piglets’ ileum or colon between the monitored experimental animal groups; Table S2: Comparison of determined cultivation counts (log CFU g^−1^) of administered bacteria present in the ileum and colon for each tested animal group; Table S3: Comparison of determined cultivation counts (log CFU g^−1^) of administered bacteria present in the ileum and colon in time; Table S4: Comparison of determined cultivation counts (log CFU g^−1^) of administered bacteria present in the ileum and colon per DPM1 and DPM2 animal groups.
