# Supplementary material for: Defined Pig Microbiota Mixture as Promising Strategy against Salmonellosis in Gnotobiotic Piglets
Source: Animals (Basel). 2024 Jun 13;14(12):1779. doi: 10.3390/ani14121779 (PMC11200913; doi:10.3390/ani14121779)
Supplement: Supplementary file 1 [file animals-14-01779-s001.zip › animals-3034410-supplementary.pdf]

Supplementary Material

Table S1: Comparison of determined cultivation counts (log CFU g^{-1}) of administered bacteria in the piglets' ileum or colon between the monitored experimental animal groups

		Ileum			Colon		
		**DPM1**	**DPM1+LT2**	**LT2**	**DPM1**	**DPM1+LT2**	**LT2**
**Total counts**	WSP	8.78 ± 0.87	**8.39 ± 0.56***	**7.57 ± 0.55***	9.60 ± 0.44	9.01 ± 0.49	8.72 ± 0.53
**Lactobacilli**	ROG	8.20 ± 0.67	7.76 ± 0.26	n.d.	9.13 ± 0.50	8.17 ± 0.86	n.d.
**Bifidobacteria**	MUP	7.73 ± 0.10	7.72 ± 0.98	n.d.	**9.38 ± 0.23***	**8.43 ± 0.77***	n.d.
	NORF	**7.56 ± 0.14***	**6.90 ± 0.63***	n.d.	8.94 ± 0.13	7.52 ± 1.05	n.d.
**Clostridia/bacilli**	WSP	4.75 ± 1.94	3.47 ± 1.94	n.d.	4.70 ± 1.09	4.58 ± 1.72	n.d.
***Salmonella* sp.**	SS	n.d.	8.27 ± 0.55	7.73 ± 0.37	n.d.	9.08 ± 0.48	8.61 ± 0.05

Cultivation counts of five bacterial groups (total counts of bacteria, lactobacilli, bifidobacteria, clostridia/bacilli, and *Salmonella* sp.) in log CFU g^{-1} were determined for three experimental animal groups and both parts of their gut, the ileum and colon. WSP – Wilkins-Chalgren agar with soya peptone; ROG – acidified Rogosa agar; MUP – acidified WSP agar with mupirocin; NORF – acidified WSP agar with mupirocin and norfloxacin; SS – *Salmonella-Shigella* agar; DPM1 – piglets associated with DPM mixture for 8 days; DPM1+LT2 – piglets associated with DPM mixture and infected with *S.* Typhimurium LT2; LT2 – piglets infected with *S.* Typhimurium LT2; n.d. – not detected. Asterisks (*) and bold font denote the statistically significant differences ($P<0.05$).

Table S2: Comparison of determined cultivation counts (log CFU g^{-1}) of administered bacteria present in the ileum and colon per each tested animal group

		DPM1		DPM1+LT2		LT2	
		**Ileum**	**Colon**	**Ileum**	**Colon**	**Ileum**	**Colon**
**Total counts**	WSP	8.78 ± 0.87	9.60 ± 0.44	8.39 ± 0.56	9.01 ± 0.49	7.57 ± 0.55	8.72 ± 0.53
**Lactobacilli**	ROG	**8.20 ± 0.67***	**9.13 ± 0.50***	7.76 ± 0.26	8.17 ± 0.86	n.d.	n.d.
**Bifidobacteria**	MUP	**7.73 ± 0.10***	**9.38 ± 0.23***	7.72 ± 0.98	8.43 ± 0.77	n.d.	n.d.
	NORF	**7.56 ± 0.14***	**8.94 ± 0.13***	6.90 ± 0.63	7.52 ± 1.05	n.d.	n.d.
**Clostridia/bacilli**	WSP	4.75 ± 1.94	4.70 ± 1.09	3.47 ± 1.94	4.58 ± 1.72	n.d.	n.d.
***Salmonella* sp.**	SS	n.d.	n.d.	**8.27 ± 0.55***	**9.08 ± 0.48***	**7.73 ± 0.37***	**8.61 ± 0.05***

Cultivation counts of five bacterial groups (total counts of bacteria, lactobacilli, bifidobacteria, clostridia/bacilli, and *Salmonella* sp.) in log CFU g^{-1} were determined for three experimental animal groups and both parts of their gut, the ileum and colon. WSP – Wilkins-Chalgren agar with soya peptone; ROG – acidified Rogosa agar; MUP – acidified WSP agar with mupirocin; NORF – acidified WSP agar with mupirocin and norfloxacin; SS – *Salmonella-Shigella* agar; DPM1 – piglets associated with DPM mixture for 8 days; DPM1+LT2 – piglets associated with DPM mixture and infected with *S.* Typhimurium LT2; LT2 – piglets infected with *S.* Typhimurium LT2; n.d. – not detected. Asterisks (*) and bold font denote

the statistically significant differences between two parts of the gut, the ileum and colon, assessed for each animal experimental group (DPM1, DPM+LT2, and LT2) separately (P<0.05).

Table S3: Comparison of determined cultivation counts (log CFU g^{-1}) of administered bacteria present in the ileum and colon in time

	Ileum		Colon	
	**DPM1**	**DPM2**	**DPM1**	**DPM2**
**Lactobacilli**	8.20 ± 0.67	7.93 ± 0.49	9.13 ± 0.50	8.95 ± 0.34
**Bifidobacteria**	7.64 ± 0.07	7.42 ± 0.21	9.16 ± 0.09	8.86 ± 0.27
**Clostridia/bacilli**	4.75 ± 1.94	4.67 ± 0.71	4.70 ± 1.09	4.33 ± 0.58

Cultivation counts of three bacterial groups (lactobacilli, bifidobacteria, and clostridia/bacilli) in log CFU g^{-1} were determined for two experimental animal groups and both parts of their gut, the ileum and colon. DPM1 – piglets associated with DPM mixture for 8 days; DPM+LT2 – piglets associated with DPM mixture and infected with *S*. Typhimurium LT2.

Table S4: Comparison of determined cultivation counts (log CFU g^{-1}) of administered bacteria present in the ileum and colon per DPM1 and DPM2 animal groups

	DPM1		DPM2	
	**Ileum**	**Colon**	**Ileum**	**Colon**
**Lactobacilli**	**8.20 ± 0.67***	**9.13 ± 0.50***	**7.93 ± 0.49***	**8.95 ± 0.34***
**Bifidobacteria**	**7.64 ± 0.07***	**9.16 ± 0.09***	**7.42 ± 0.21***	**8.86 ± 0.27***
**Clostridia/bacilli**	4.75 ± 1.94	4.70 ± 1.09	4.67 ± 0.71	4.33 ± 0.58

Cultivation counts of three bacterial groups (lactobacilli, bifidobacteria, and clostridia/bacilli) in log CFU g^{-1} were determined for two experimental animal groups and both parts of their gut, the ileum and colon. DPM1 – piglets associated with DPM mixture for 8 days; DPM2 – piglets associated with DPM mixture for 14 days. Asterisks (*) and bold font denote the statistically significant differences between two parts of the gut, the ileum and colon (P<0.05).
